# Plant buffering against the high-light stress-induced accumulation of *CsGA2ox8* transcripts via alternative splicing to finely tune gibberellin levels and maintain hypocotyl elongation

**DOI:** 10.1038/s41438-020-00430-w

**Published:** 2021-01-01

**Authors:** Bin Liu, Shuo Zhao, Pengli Li, Yilu Yin, Qingliang Niu, Jinqiang Yan, Danfeng Huang

**Affiliations:** 1 School of Agriculture and Biology, Shanghai Jiao Tong University, Key Laboratory of Urban Agriculture (South), Ministry of Agriculture, Dongchuan Road, Shanghai, 200240 China; 2 Department of Plant Genomics, Centre for Research in Agricultural Genomics (CRAG), CSIC-IRTA-UAB-UB, Bellaterra, 08193 Spain; 3 Vegetable Research Institute, Guangdong Academy of Agricultural Sciences, Guangzhou, 510640 China; 4 Guangdong Key Laboratory for New Technology Research of Vegetables, Guangzhou, 510640 China

**Keywords:** Light responses, RNA splicing

## Abstract

In plants, alternative splicing (AS) is markedly induced in response to environmental stresses, but it is unclear why plants generate multiple transcripts under stress conditions. In this study, RNA-seq was performed to identify AS events in cucumber seedlings grown under different light intensities. We identified a novel transcript of the gibberellin (GA)-deactivating enzyme *Gibberellin 2-beta-dioxygenase* 8 (*CsGA2ox8*). Compared with canonical *CsGA2ox8.1*, the *CsGA2ox8.2* isoform presented intron retention between the second and third exons. Functional analysis proved that the transcript of *CsGA2ox8.1* but not *CsGA2ox8.2* played a role in the deactivation of bioactive GAs. Moreover, expression analysis demonstrated that both transcripts were upregulated by increased light intensity, but the expression level of *CsGA2ox8.1* increased slowly when the light intensity was >400 µmol·m^−2^·s^−1^ PPFD (photosynthetic photon flux density), while the *CsGA2ox8.2* transcript levels increased rapidly when the light intensity was >200 µmol·m^−2^·s^−1^ PPFD. Our findings provide evidence that plants might finely tune their GA levels by buffering against the normal transcripts of *CsGA2ox8* through AS.

## Introduction

Gibberellins (GAs) are known to be the dominant hormone governing hypocotyl elongation^1,2^. GAs regulate cell expansion, thus causing changes in hypocotyl length or plant height via the GA signaling pathway. GA-GID1-DELLA is a widely accepted signaling module through which bioactive GAs regulate plant growth and development^2^. For example, GIBBERELLIN INSENSITIVE DWARF 1 (GID1)-inactivation mutants show reduced plant height^3,4^, and substitution of proline to leucine in *DS-3*, which encodes a DELLA protein, results in semi-dwarf oilseed rape plants^5^.

Light is among the most essential factors for plant growth and development^6^. Seedlings grown under dark conditions are characterized by relatively long hypocotyls compared with those grown under light conditions^7,8^. One of the reasons for this is that under light conditions, the GA level decreases, while under dark or shaded conditions, the GA level increases^9–11^. Most related studies have focused mainly on low-light (shading)-induced GA biosynthesis for signaling^12^, and only a few studies have shown high-light-induced excessive GA deactivation. However, it remains unknown how plants respond to high-light stress and finely tune their GA levels to maintain growth.

Alternative splicing (AS) is a widespread mechanism in eukaryotes in which different protein variants are generated from a single gene via different combinations of splicing sites. AS is a prevalent approach in which plants respond to environmental stress, but it is counterintuitive why plants would invest in protein synthesis under conditions of declining energy supplies^13,14^. Chaudhary et al. proposed that, under stress conditions, plants buffer against normal protein synthesis levels via AS to decrease the translation of a significant portion of the transcriptome and produce the protein isoforms necessary for adaptation to stresses; however, the mechanisms of this process are not yet known^14^.

In this study, we performed RNA-seq to identify differentially expressed genes and AS events in cucumber (*Cucumis sativus* L.) seedlings grown under different light intensities. We focused on GA-related genes and found a novel transcript of *Gibberellin 2-beta-dioxygenase* (*CsGA2ox8*), *CsGA2ox8.2*, with intron retention (IR) between the second and third exons. *Gibberellin 2-beta-dioxygenases* compose a family whose members can catalyze the deactivation of bioactive GAs or their precursors^15^. Most studies have shown that increasing transcripts of *GA2ox*s can reduce plant height by regulating endogenous bioactive GA levels^15–20^. We found that under high-light stress, cucumber seedlings tended to generate more nonfunctional *CsGA2ox8.2* but not functional canonical *CsGA2ox8.1*. In other words, the plants buffered against normal transcript levels of *CsGA2ox8* via AS to limit the inactivation process of GAs under high-light stress, and this strategy may allow seedlings to maintain a certain GA level and sustain hypocotyl elongation.

## Results and discussion

### *CsGA2ox8* expression is upregulated in response to increasing light intensity

**Fig. 1:**
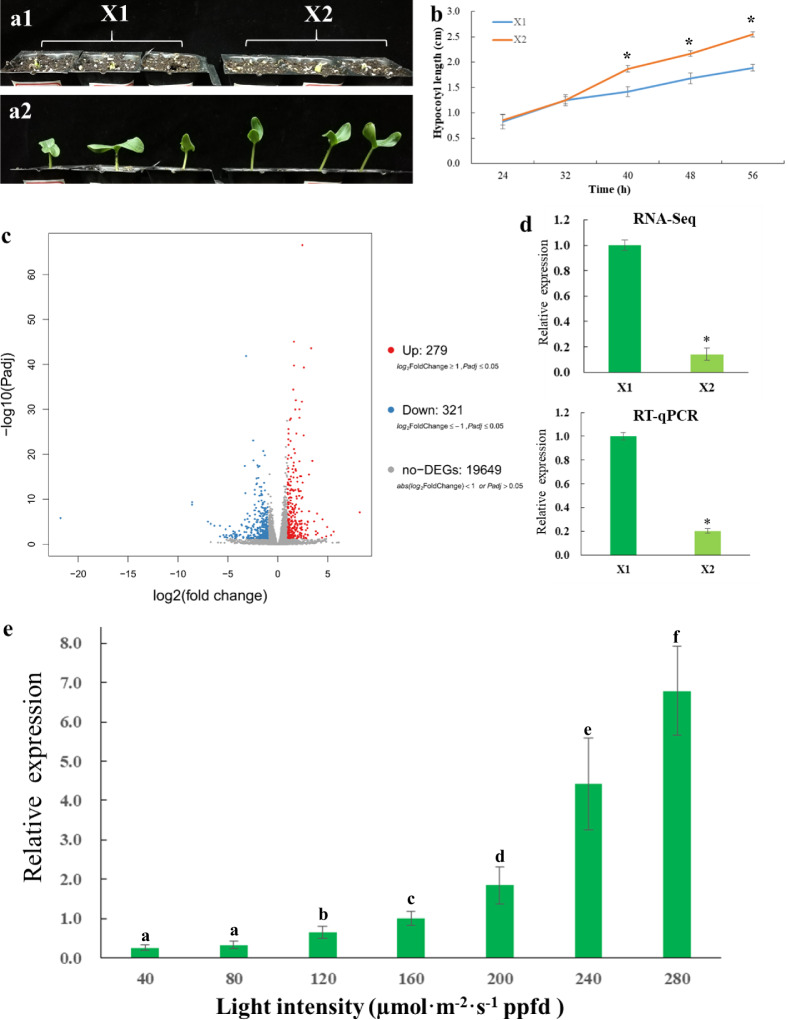
Expression of *CsGA2ox8* is positively regulated by light intensity. **a** Phenotypes of cucumber seedlings before (**a1**) and after (**a2**) treatment with fluorescent lights at two intensities (X1, 180 µmol·m^−2^·s^−1^ PPFD; X2, 40 µmol·m^−2^·s^−1^ PPFD) for 56 h. **b** Hypocotyl lengths of cucumber seedlings between the two treatments. The error bars indicate the standard deviations (SDs; *n* = 5). The asterisks indicate significant differences in hypocotyl lengths between X1 and X2 (*P* < 0.05). **c** Volcano plot of the transcriptome between X1- and X2-treated cucumber seedlings. **d** Quantitative reverse-transcription PCR results (three replications) validating the different expressions of *CsGA2ox8* between the two treatments. The asterisks indicate significant differences in gene expression between X1 and X2 (*P* < 0.05). **e** Relative expression of *CsGA2ox8* under different light intensities. The lowercase letters indicate that *CsGA2ox8* transcription was modified by light intensity (*P* < 0.05, three replications)

We recorded the changes in cucumber hypocotyl lengths after the cotyledons emerged from the soil under treatments involving two light intensities: X1 (180 µmol·m^−2^·s^−1^ PPFD) and X2 (40 µmol·m^−2^·s^−1^ PPFD). The hypocotyl lengths of seedlings grown under treatment X1 were shorter than those grown under treatment X2 (Fig. 1a), and a significant difference was observed after 40 h of light treatment (Fig. 1b). To investigate the molecular differences of seedlings grown under the two light conditions, a transcriptome analysis was performed. In total, 279 upregulated and 321 downregulated genes were identified between the two treatments (Fig. 1c, Supplementary Table S1). In this study, we focused on GA-related genes because bioactive GAs have a large effect on hypocotyl elongation in cucumber^1^. We found that *CsGA2ox8*, a gene whose product catalyzes the deactivation of bioactive GAs, was significantly upregulated in treatment X1 compared to treatment X2. This expression difference was confirmed by quantitative reverse-transcription PCR (qRT-PCR) (Fig. 1d). These results suggested that the expression of *CsGA2ox8* might be affected by light intensity. To test this hypothesis, we treated young cucumber seedlings with seven different light intensities (40, 80, 120, 160, 200, 240, and 280 µmol·m^−2^·s^−1^ PPFD) and quantified the expression of *CsGA2ox8* in seedlings grown under these conditions. As shown in Fig. 1e, the transcript level of *CsGA2ox8* increased with increasing light intensity. Pioneering studies have shown that light modulates photomorphogenesis-related hypocotyl elongation mainly by interacting with GAs, and many genes are involved in this light-GA system^10^. Our results suggested that the expression of the GA deactivation factor *CsGA2ox8* was upregulated by increased light levels, which might be involved in light-regulated hypocotyl elongation.

### Differential AS (DAS) analysis for identifying novel transcripts of *CsGA2ox8*

**Fig. 2:**
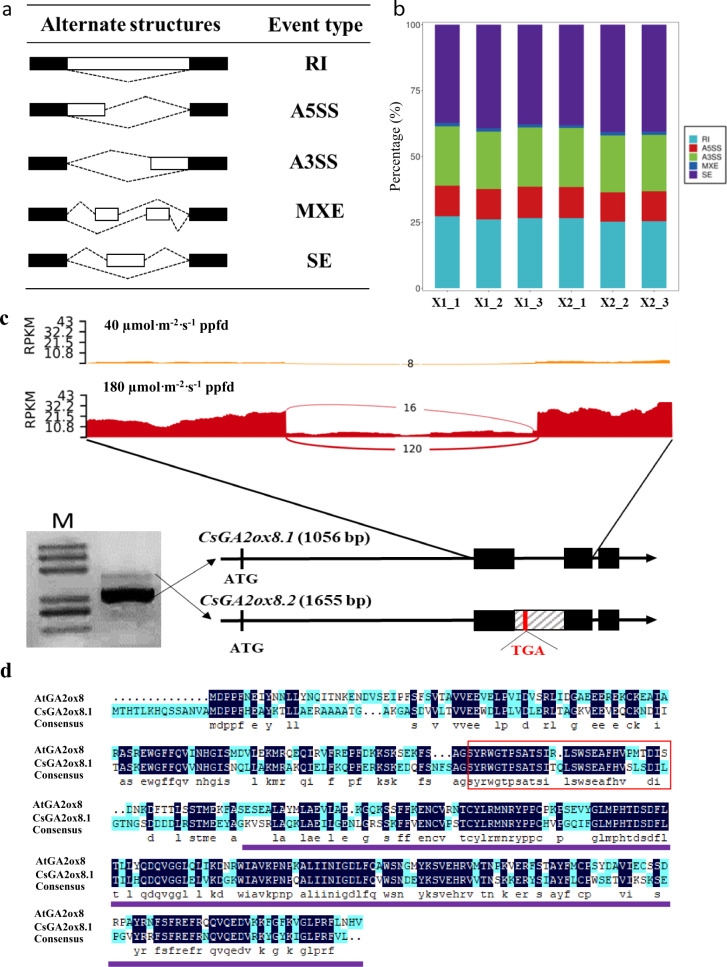
Analysis of differential alternative splicing (DAS) for the identification of novel transcripts of *Gibberellin 2-beta-dioxygenase 8 (CsGA2ox8)*. **a** Identification of five AS events in the RNA-seq datasets of cucumber seedlings grown under two light conditions. RI, retained intron; A5SS, alternative 5′ splice site; A3SS, alternative 3′ splice site; MXE, mutually exclusive exon; and SE, skipped exon. Black boxes, exons flanking AS events; white boxes, differentially included exons; dotted lines, sequences spliced together. **b** Stacked bars showing five AS events and their frequency in each replication under the two light conditions. **c** Validation of the intron retention (IR) of *CsGA2ox8* via reverse-transcription PCR (RT-PCR). A termination codon, TGA (red line), was detected in the retained intron (gray box). M, Trans2K Plus DNA Marker. **d** Alignment of the amino acid sequences of AtGA2ox8 and CsGA2ox8.1. The unique conserved domain of GA2ox8.1 is denoted by the red box above. Purple line, the lost amino acid region of CsGA2ox8.2

AS is a prevalent approach used by plants in response to stress and involves the generation of multiple transcripts from the same gene^21^. In this study, five types of AS events, namely, RIs (retained introns), A5SSs (alternative 5′ splice sites), A3SS (alternative 3′ splice sites), MXEs (mutually exclusive exons), and SEs (skipped exons), were identified by RNA-seq analysis (Fig. 2a, Supplementary Table S2). Among them, SEs and RIs accounted for more than 25% of all AS events (Fig. 2b), which is similar to that reported in *Arabidopsis*^22,23^.

Among all RI events, we found a novel transcript of *CsGA2ox8* with an IR between the second and third exons. The original transcript of *CsGA2ox8* was defined as *CsGA2ox8.1*, and the isoform with IR was defined as *CsGA2ox8.2* (Fig. 2c). To validate the existence of *CsGA2ox8.2*, the coding regions of *CsGA2ox8* of plants under treatment X2 were amplified and then sequenced. Two distinct bands were observed after RT-PCR-based amplification (Fig. 2c). After sequencing, the two amplicons were found to be 1056 and 1655 bp in length (Fig. 2c). Sequence comparison showed the same result as that after RNA-seq, and a schematic diagram of this event is illustrated in Fig. 2c. RIs promoted the disruption of the open reading frame of *CsGA2ox8*, resulting in an mRNA with a premature stop codon (TGA), and leading to a truncated protein (Fig. 2c). Compared with other GA2ox proteins in *Arabidopsis*, the AtGA2ox7 and AtGA2ox8 proteins have a unique region^15^. Alignment of the amino acid sequences of AtGA2ox8 and CsGA2ox8.1 revealed that the CsGA2ox8.1 protein had a conserved motif (Fig. 2d, denoted by a red box), which may define the specificity of the reactions catalyzed by these enzymes^15^. The absence of amino acids in CsGA2ox8.2 is indicated using a purple line in Fig. 2d.

### Overexpression of *CsGA2ox8.1* but not *CsGA2ox8.2* decreases GA levels and the length of the hypocotyl in *Arabidopsis thaliana*

**Fig. 3:**
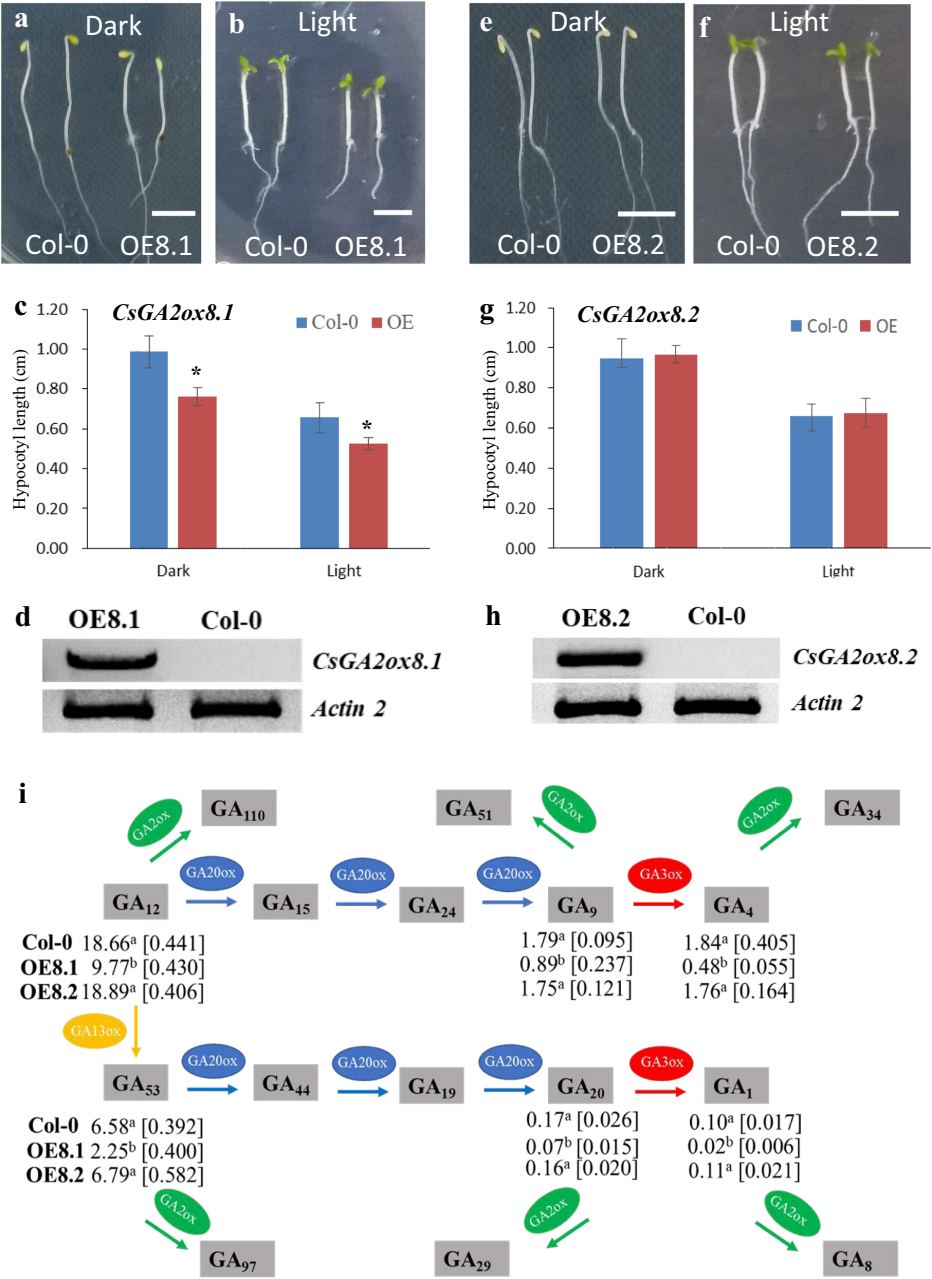
*CsGA2ox8.1* but not *CsGA2ox8.2* had a role in the deactivation of bioactive gibberellins (GAs). **a**, **b** *CsGA2ox8.1* overexpression (OE8.1) lines had shortened hypocotyls under both dark and light conditions. Scale bars, 5 mm. **c** Hypocotyl lengths of the indicated genotypes in (**a**) and (**b**). The error bars indicate the standard deviations (SDs, *n* > 20). The asterisks indicate significant differences between the hypocotyl lengths of OE8.1 lines and Col-0 (*P* < 0.05). **d** *GA2ox8.1* is overexpressed in OE8.1 but is not expressed in Col-0. **e**, **f** *CsGA2ox8.2* overexpression (OE8.2) lines had hypocotyl lengths similar to those of Col-0 under both dark and light conditions. **g** Hypocotyl lengths of the indicated genotypes in (**e**) and (**f**). The error bars indicate SDs (*n* > 20). **h** *GA2ox8.2* is overexpressed in OE8.2 but is not expressed in Col-0. **i** Profile of endogenous GAs in Col-0, OE8.1, and OE8.2 lines

*GA2ox* genes function in reducing GAs^15–20,24^. Given that *CsGA2ox8.2* lost the third and fourth exons from *CsGA2ox8.1* (Fig. 2c, d), we questioned whether *CsGA2ox8.2* still maintains the same function as that of *CsGA2ox8.1*. To verify this, *CsGA2ox8.1* and *CsGA2ox8.2* were overexpressed in *Arabidopsis* Col-0. *CsGA2ox8.1* overexpression lines (OE8.1) had shorter hypocotyl lengths than Col-0 under both light and dark conditions (Fig. 3a–c). The RT-PCR results suggested that *GA2ox8.1* was overexpressed in OE8.1 but not in Col-0 (Fig. 3d), suggesting that *GA2ox8.1* was functional for hypocotyl elongation. However, the hypocotyl length of *CsGA2ox8.2* overexpression lines (OE8.2) was not significantly different from that of Col-0 (Fig. 3e–g), even though the transcript level of *GA2ox8.2* was as high as that of *GA2ox8.1* (Figs. 3d, h). Next, the GA levels were quantified in the Col-0, OE8.1, and OE8.2 lines. Based on the GA synthetic pathway, *GA2ox* genes encoding enzymes that convert GA_12_, GA_9_, GA_53_, GA_20_, GA_1_, and GA_4_ to GA_110_, GA_51_, GA_97_, GA_29_, GA_8_, and GA_34_, respectively^25,26^ (Fig. 3i). In this study, we found that GA_12_, GA_9_, GA_53_, GA_20_, GA_1_, and GA_4_ significantly decreased in OE8.1 compared with Col-0, and no significant differences in GA levels were detected between OE8.2 and Col-0 (Fig. 3i), suggesting that *CsGA2ox8.1* is a functional transcript but that *CsGA2ox8.2* is not.

AS is a prevalent approach in which plants respond to environmental stress. One hypothesis is that plants buffer against normal protein synthesis levels via AS to decrease the translation of a significant portion of the transcriptome under stress conditions^14^. Therefore, for future studies, it is important to know how CsGA2ox8.1 and CsGA2ox8.2 work at the protein level.

The homolog of *CsGA2ox8* in *Arabidopsis* is *AtGA2ox8*, whose product functions in hydroxylating the C_20_-GA precursors GA_12_ and GA_53_^15^. In this study, consistent with the results from overexpression of *AtGA2ox8*^15^, GA_12_ and GA_53_ levels dramatically decreased in OE8.1, suggesting that *CsGA2ox8* has a function similar to that of *AtGA2ox8*. However, we found that the content of GA_9_, GA_20_, GA_1_, and GA_4_ also decreased, which is inconsistent with the results of Schomburg et al. (2003), who reported that GA_9_, GA_20_, and GA_4_ levels increased in *AtGA2ox8* overexpression lines^15^. GA_12_ is the precursor of GA_9_ and GA_4_, and GA_53_ is the precursor of GA_20_ and GA_1_^25^. Therefore, it is possible that decreasing GA_12_ and GA_53_ levels would affect the synthesis of GA_9_, GA_4_, GA_20_, and GA_1_. Taken together, these results suggest that *CsGA2ox8.1*, rather than its *CsGA2ox8.2* isoform, regulated hypocotyl elongation, and this regulation was achieved mostly via decreasing endogenous GA levels.

### Plants tend to generate nonfunctional *CsGA2ox8.2* but not functional *CsGA2ox8.1* under high-light stress

As one type of AS, IR mostly results in the production of nonfunctional transcripts carrying premature termination codons targeted for degradation via the nonsense-mediated mRNA decay pathway^27,28^. However, it is unclear why plants perform AS under stress conditions. Given that *CsGA2ox8.2* was detected under relatively high-light conditions and that *CsGA2ox8.2* seemed to be a nonfunctional transcript, we hypothesized that *CsGA2ox8.2* was generated for buffering against normal *CsGA2ox8.1* transcripts under high-light stress to finely tune GA levels and maintain plant growth. Chaudhary et al. posited the same idea and proposed that, under stress conditions, plants buffer against normal protein synthesis levels via AS to decrease translation of a significant portion of the transcriptome, but the mechanisms of this process have not yet been elucidated^14^.

**Fig. 4:**
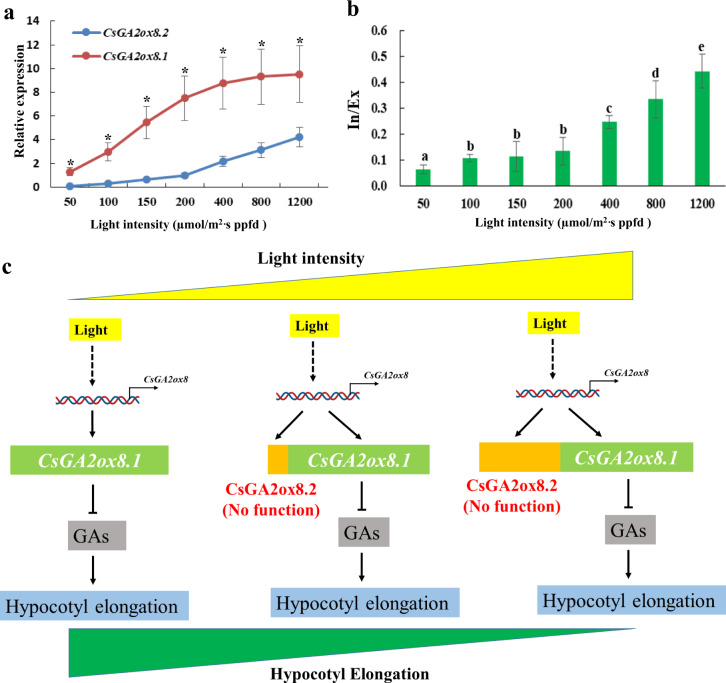
Plants tend to generate more *CsGA2ox8.2* but not *CsGA2ox8.1* under high-light stress. **a** Relative expression levels of *CsGA2ox8.1* and *CsGA2ox8.2* under different light conditions as measured via quantitative reverse-transcription PCR (three replications). The asterisks indicate significant differences in gene expression between *CsGA2ox8.1* and *CsGA2ox8.2* (*P* < 0.05). **b** High light promotes the expression of *CsGA2ox8.2*. The RNA products are quantified via the *CsGA2ox8.2/CsGA2ox8.1* [inclusion/exclusion (In/Ex)] ratio. **c** Model depicting that plants buffer against high-light stress-induced functional CsGA2ox8.1 via alternative splicing of a nonfunctional CsGA2ox8.2 isoform to finely tune gibberellin (GA) levels and maintain hypocotyl elongation

To test our hypothesis, the relative abundance of the two mRNA forms of *CsGA2ox8* in plants under 50, 100, 150, 200, 400, 800, and 1200 µmol·m^−2^·s^−1^ PPFD was analyzed by RT-PCR. The results showed that both transcripts were positively regulated by light, and *CsGA2ox8.1* was the dominant transcript under all light conditions (Fig. 4a). However, the expression level of *CsGA2ox8.1* increased slowly when the light intensity was >400 µmol·m^−2^·s^−1^ PPFD, while that of *CsGA2ox8.2* increased rapidly when the light intensity was >200 µmol·m^−2^·s^−1^ PPFD (Fig. 4a). Furthermore, the inclusion/exclusion (In/Ex) ratio (*CsGA2ox8.2*/*CsGA2ox8.1*) significantly increased when the light intensity was >400 µmol·m^−2^·s^−1^ PPFD (Fig. 4b). In other words, the *CsGA2ox8.2* expression level was negligible under low-light conditions, but under increasing light intensity, especially that corresponding to high-light stress, plants tended to generate more nonfunctional *CsGA2ox8.2* transcripts and limit the increase in functional *CsGA2ox8.1* levels (Fig. 4c). In this way, plants could produce less functional *GA2ox* and could maintain a certain level of GA for hypocotyl elongation under high-light conditions.

AS is known to promote plant stress tolerance^13^. For example, stress imposed by high salinity promotes the use of noncanonical splice sites^29^. In addition, AS events finely tune gene expression by regulating the ratio of active and nonactive isoforms^30^. Similarly, we showed that high-light stress increased the *CsGA2ox8.2*/*CsGA2ox8.1* ratio in this study, suggesting a new mechanism for the GA-deactivating-enzyme response to high-light stress. However, it is not clear how high light modulates AS events. To our knowledge, AS events are mediated by core spliceosomal components (small nuclear ribonucleoproteins) as well as RNA-binding proteins (serine/arginine-rich proteins and heterogeneous nuclear ribonucleoproteins)^30^. One hypothesis is that high-light stress affects the expression or protein structure of spliceosome genes, thereby modulating AS events at the whole-genome level.

Our results suggest that IR may have been employed to finely tune gene expression by altering the ratio between functional and nonfunctional variants. Following a BLAST search of The Arabidopsis Information Resource (TAIR, https://www.arabidopsis.org/) and the MSU Rice Genome Annotation Project Database (http://rice.plantbiology.msu.edu/index.shtml), AT4G21200 and LOC_Os02g41954 were identified has sharing high sequence homology (57.42% and 49.03%, respectively) with that of CsGA2ox8. As shown in TAIR, *AT4G21200* has one splice variant with an IR between the second and third exons, which is the same as *CsGA2ox8.2*, with the RI promoting the disruption of the open reading frame of *AT4G21200*, resulting in the generation of an mRNA with a premature stop codon (TGA), leading to a truncated protein^23^. From the rice database, *LOC_Os02g41954* also has one splice variant, which has a premature stop codon (TGA) between the second and third exons^30^. These findings suggest that AS of *GA2ox8* commonly occurs in plants, and this strategy might also be used by other plant species to buffer against the stress-responsive transcriptome and promote efficient adaptation to stress conditions.

## Materials and methods

### Plant materials and growth conditions

Cucumber inbred line 9930 was used in this study. Seeds were surface sterilized and soaked in water for 3 h, after which they were then transferred to Petri dishes lined with wet filter papers and germinated at 28 °C. The germinated seeds were then sown in autoclaved soil at 24 °C. Single cucumber seedlings with cotyledons emerging from the soil were chosen for light treatment. *A. thaliana* Col-0 was used in this study. The seeds were disinfected and germinated on Murashige–Skoog (MS) media consisting of 1% sucrose and 0.2% Phytagar at 4 °C for 3 days before being moved into a growth chamber at 22 °C^31^.

### Light treatment and hypocotyl measurements

Light intensities were measured using a Lighting Passport^TM^ ALP-01 Handheld Spectrometer (HESON, Shanghai, China). Cucumber seedlings were grown in a greenhouse with “DE-HPS 1000 W” lights. To maintain the light conditions within an appropriate range, the plants were placed at different heights below the fixtures. For example, to generate a high-light condition, the plants were moved closer to the lamps; to generate low-light conditions, the plants were moved farther from the lamps or were covered with film. For *Arabidopsis*, the plates with disinfected seeds were treated at 4 °C for 72 h and then placed under white light for 10 h to induce uniform germination. Afterward, the plates were transferred to dark or light conditions and then incubated at 22 °C for 3–6 days for hypocotyl measurements. The hypocotyl lengths of the seedlings were measured using a tapeline.

### GA level analysis

The GA level was analyzed as previously reported, with moderate changes^32^. Briefly, for product purification, ~0.5 g of the sample was quickly immersed in liquid nitrogen, homogenized, and extracted with 5 mL of precooled 80% methanol for 20 h at 4 °C. Afterward, the samples were shaken at 4 °C for 30 min and then centrifuged at 18,000 × *g* for 20 min at 4 °C. The supernatant was collected and immersed in liquid nitrogen. Then, the samples were dissolved in 100 µL of chromatography-grade methanol supplemented with 0.1 M glacial acetic acid and filtered through a 0.22 µm filter. High-performance liquid chromatography-based analysis was performed using an Agilent (CA, USA) Zorbax SB-C_18_ column (5 µm, 4.6 × 250 mm). The mobile phase A solvent consisted of 100% methanol, and mobile phase B was 0.1 M acetic acid. The gradient elution was performed as follows: 0–40 min, 3% A and 97% B; after 40 min, 67.6% A and 32.2% B. The flow rate was 1 mL·min^−1^, and the column temperature was set at 30 °C. Chromatograms were quantified using the Agilent chromatography workstation. Each sample involved three replicates from independent experiments.

### RNA-seq analysis and validation

Cucumber seedlings were subjected to two light conditions (X1, 180 µmol·m^−2^·s^−1^ PPFD; X2, 40 µmol·m^−2^·s^−1^ PPFD) for 56 h, after which the hypocotyls were harvested for RNA extraction (SV Total RNA Isolation System, Promega). Each treatment was replicated three times. The RNA quality was measured using an Agilent 2100 Bioanalyzer system (Agilent Technologies, CA, USA), with a RIN/RQ (RNA integrity number/RNA quality index) ≥8. Sequencing libraries were prepared using a NEBNext^®^ Ultra^TM^ RNA Library Prep Kit for Illumina^®^ (NEB, USA). The libraries were sequenced on a HiSeq 4000 platform (Illumina, San Diego, CA). Thereafter, unknown or low-quality reads, as well as reads with adapters, were passed through a filter (SOAPnuke v1.5.2, https://github.com/BGI-flexlab/SOAPnuke) to generate clean reads^33^. The remaining high-quality clean reads were mapped to the Cucumber Reference Genome V3 version (ftp://cucurbitgenomics.org/pub/cucurbit/genome/cucumber/Chinese_long/v3/)^34^ using Bowtie2 v2.2.5^35^. Differential expression analyses were subsequently carried out using RSEM v1.2.12^36^ software. Differentially expressed genes were defined as those having an |log2 fold change (FC) | ≥ 1 and a *q*-value ≤0.05. DAS analyses were performed using rMATS v3.2.5^37^. Transcripts were deemed to be the result of DAS when the false discovery rate was ≤0.05.

To validate the *GA2ox8* AS isoforms, seedlings were treated with the same light conditions as those applied for RNA-seq (X1, 180 µmol·m^−2^·s^−1^ PPFD; X2, 40 µmol·m^−2^·s^−1^ PPFD) for 56 h, after which the hypocotyls were harvested for RNA extraction, cDNA synthesis, and PCR-based analysis. The primers used are listed in Supplementary Table 3.

### Sequence alignment

Sequence alignments were performed based on the amino acid sequences of CsGA2ox8.1, CsGA2ox8.2, and AtGA2ox8. Multiple sequence alignment was performed using DNAMAN for Windows (Lynnon Corporation, Quebec).

### Gene expression analysis

Gene expression analysis methods were used as previously reported^31^. Total RNA was extracted from the hypocotyls using an SV Total RNA Isolation System (Promega), and cDNA was synthesized using a MultiScribe^TM^ Reverse Transcriptase Kit (Applied Biosystems) according to the manufacturer’s instructions. Quantitative RT-PCR was performed using a SYBR^®^ Premix *Ex Taq*^TM^ Kit (TaKaRa, China) in conjunction with an Applied Biosystems 7500 Real-time PCR system. For RT-PCR, PCR primers were designed to amplify two isoforms of different sizes, and their RNA products were quantified and reported as an In/Ex ratio^38^. Cucumber *α-TUBULIN* (*TUA*) and *Arabidopsis ACTIN 2* were used as internal controls^39^. The primers used are listed in Supplementary Table S3.

### Transformation of *A. thaliana*

Transformation assays were performed as previously described^31^. To generate *CsGA2ox8.1* and *CsGA2ox8.2* overexpression constructs, full-length *CsGA2ox8.1* and *CsGA2ox8.2* cDNAs were cloned and inserted into a pBI121 vector between the XbaI and SmaI restriction sites^40^. The constructs were then introduced into *Agrobacterium tumefaciens* strain LBA4404 by electroporation, which were then transformed into wild-type *A. thaliana* (Col-0), as previously described^41^. The transgenic plants were screened on MS media supplemented with 50 mg/L kanamycin and then confirmed via PCR. The primers used are listed in Supplementary Table S3.

### Statistical analysis

All the treatments mentioned in this study involved at least three independent biological and technical replicates. The results were analyzed using analysis of variance. All the analyses were carried out using Statistics Analysis System 15.1 for Windows.

## Supplementary information

Supplementary Table S1

Supplementary Table S2

Supplementary Table S3
